# Quantifying the nutritional and income loss caused by crop raiding in a rural African subsistence farming community in South Africa

**DOI:** 10.4102/jamba.v13i1.1040

**Published:** 2021-11-30

**Authors:** Tlou D. Raphela, Neville Pillay

**Affiliations:** 1 Disaster Management Training and Education Centre for Africa, University of the Free State, Bloemfontein, South Africa; 2 School of Animal, Plant and Environmental Sciences, Faculty of Science, University of the Witwatersrand, Johannesburg, South Africa

**Keywords:** crop raiding, economic loss, energy loss, maize, mitigation measures

## Abstract

Globally, crop damage by wildlife contributes to food insecurity through the direct loss of food and income. We investigated the calories lost and the potential economic impact of crop raiding at subsistence homesteads abutting the Hluhluwe Game Reserve, and assessed mitigation measures to combat crop raiding. We quantified the seasonal loss of calories (kJ/g) of four common crops, namely, beetroot, common bean, maize, and spinach, and determined the seasonal potential income loss. We used a stratified sampling approach to sample the homesteads. We found that season, crop type and the interaction between season and crop type predicted relative calorie loss and potential income loss, with the highest income loss recorded for spinach in the dry season. Significant differences were found for the potential income loss for all crop types in the wet season, and for the interaction between the crop types (maize, spinach) and the wet season. Farm slope was also a significant predictor of the relative calorie loss. Crop raiding animals, crops raided and distance of farms from the reserve all had a significant effect on the choice of mitigation measures of farmers. The highest relative calorie loss was for maize during the dry season, which could affect the subsistence farmers by reducing their daily calorie intake. This has an impact on their food security, especially during the dry season. Moreover, the most preferred mitigation measure used by farmers can have opportunity costs. These results have important implications for food security policies and practices.

## Introduction and background

Crop raiding is one of the most complex and challenging socio-environmental problem facing the world today (García-Frapolli et al. 2018). The impact of crop raiding is predicted to greatly affect vulnerable subsistence farmers abutting protected areas (L’Roe & Naughton-Treves 2017). Crop raiding can radically influence the nutrition of communities, particularly poor rural farmers (Graham et al. 2007), often leading to food insecurity (Raphela & Pillay 2021). Thus, impoverished rural communities often face the twin challenges of food shortages and crop raiding (Gontse, Mbaiwa & Thakadu 2018). This is particularly true for many African subsistence communities (Gloriose 2019; Gontse et al. 2018). Extensive damage through crop raiding can impact the farmers’ lives and livelihoods (Hill 2017b), compromise their food security (Ango, Börjeson & Senbeta 2017) and reduce their tolerance of wildlife (Tiller 2021).

Here, we documented the nutritional impact and income loss of a rural African subsistence farming community as a result of crop raiding. Such communities are marginalised in the crop raiding literature despite their vulnerabilities. Vulnerability in this context is the extent to which the farms are susceptible to the adverse effects of crop raiding (González et al. 2017). Crop loss because of crop raiding by wildlife has been reported worldwide (Alemayehu & Tekalign 2020; Hill 2018; Seoraj-Pillai & Pillay 2016) as one of the risks faced by farmers (Siljander et al. 2020). In Southern Africa, elephants and primates are the main crop-raiders, particularly of maize (*Zea mays*; Hill 2017; Troup et al. 2020). We found little evidence in the literature about the nature or extent of damage in South Africa, apart from the finding by Seoraj-Pillai and Pillay (2016) that South Africa has more Human-Wildlife Conflict (HWC) cases than developed countries. Although some farmers experience near-total crop destruction, most might experience medium- to low-level damage (Quandt 2021; Siljander et al. 2020).

Worldwide, subsistence farmers have expressed their concerns about food shortage as a result of raiding by wildlife (Nyirenda et al. 2018; Weinmann 2018). Crop raiding reduces the amount of food available to a subsistence homestead (Maurice et al. 2019), thereby diminishing their daily nutritional intake (Mkanda 1994; Nahonyo 2001; Naughton-Treves et al. 1998). Quandt (2021) reported that crop raiding can contribute significantly to changes in peoples’ diets leading to an increased dependency on purchased items and a decline in overall nutrition. Indeed, Jiao (2019) reported that rural populations near protected areas have a lower nutritional status as a result of a significantly lower agricultural yield, which is partly because of crop raiding.

Crop raiding influences the nutritional quality of diets indirectly (Sitati, Walpole & Leader-Williams 2005). Barirega et al. (2010) found that homesteads adjacent to the Queen Elizabeth National Park in Uganda that experienced crop raiding had a lower dietary diversity index (i.e. ‘a qualitative measure of food consumption that reflects household access to a variety of foods, and is also a proxy for nutrient adequacy of the diet of individuals’; De Oliveira Otto et al. 2015:3). Furthermore, cash crops are damaged which would provide income for food to supplement existing diets (Boyd et al. 1999; Nahonyo 2001).

The economic impact of crop raiding on farming has been variously recorded in several studies (Ango et al. 2017; Mc Guinness & Taylor 2014; Yang et al. 2020). For example, in Rwanda, Mc Guinness and Taylor (2014) reported substantial crop losses and replacement costs possibly reaching 10% – 20% of total household income because of crop raiding by forest dwelling primates.

Overall, crop losses because of raiding by wildlife impacts the food security, especially of subsistence farmers adjacent to the protected areas (Hill 2018). The meaning of food security is not always obvious (Kiffner et al. 2021). The Food and Agricultural Organisation (FAO 1996) defines food security as:

The situation that exists when all people, at all times, have physical, social and economic access to sufficient, safe and nutritious food that meets their dietary needs and food preferences for an active and healthy life. (p. 12)

Using this definition, we assumed that crop raiding will primarily impact the quantity of food available, thereby leading to food insecurity.

Rural communities depend on subsistence farming for their living. Thus, when there are disastrous events such as droughts and crop raiding, these rural poor people are adversely affected by these events. (Newsham & Thomas 2009). As these communities depend directly on this food production system for their survival, there are profound implications for the security of their livelihoods (Ziervogel et al. 2006). Farmers have to develop coping strategies to reduce the risks (Davies et al. 2009).

Global crises are aggravating the risks already faced by the poor and vulnerable people in rural areas, particularly subsistence farmers (Davies et al. 2009). With climate change, the magnitude and frequency of the problems faced by subsistence farmers are always changing. Thus, there should be efforts to investigate and quantify the costs of crop raiding suffered by subsistence farmers in order to apply relevant risk reduction measures. These measures seek to mitigate the risks faced by poor people and make their livelihoods more resilient to the impacts of agricultural loss (Davies et al. 2009). In agriculture, risk reduction programmes have been used to lessen the effects of persistent food shortages and prevent widespread famines (Schipper & Pelling 2006). The impacts of crop raiding can lead to increased vulnerability of poor people and a downward spiral of poverty (Setchell et al. 2021).

Mitigation is part of risk reduction, and plays an important part in sustainable livelihoods (Russell-Smith et al. 2017). Scarecrows and traditional crop guarding are considered to be important mitigation measures to deter crop-raiding animals in subsistence communities (Megaze, Balakrishnan & Belay 2017). These communities are impoverished and rely heavily on the crops they grow for subsistence, and most of them cannot afford effective mitigation measures, such as exclusive fencing used on commercial farms (Ngama et al. 2018). In fact, very little has been written about subsistence farmers’ knowledge of crop pests, the impact of pests on standing crops and the existing pest mitigation measures (Dent & Binks 2019). Yet, this information is of importance in HWC studies (Altieri 2019).

South Africa has been reported to have more HWC cases than developed countries such as Australia and North America (Seoraj-Pillai & Pillay 2017). In addition, HWC because of competition for shared natural resources between people and wild animals influences the food security of people and the well-being of people and animals especially in South Africa (Nieman, Wilkinson & Leslie 2020). Furthermore, communities adjacent to the protected areas often exploit natural resources because of poverty, thus bringing the communities into conflict with wild animals and protection area authorities in South Africa (Swemmer, Mmethi & Twine 2017). Moreover, the global assessment showed that people in developing countries are vulnerable to HWC, but Grey et al. (2017) reported that HWC can be reduced by overcoming the mismatch between actual and perceived levels of damages in a study around Soutpansberg Mountains in South Africa. Therefore, to address these shortcomings, we quantified nutritional and income loss because of crop raiding in a rural African subsistence farming community adjacent to the Hluhluwe Game Reserve in South Africa, by direct evaluation of calories lost and also by gauging farmers’ opinions. Furthermore, we investigated the mitigation measures employed by this community to combat crop raiding. We did not consider post-harvest loss because none of the farmers stored food during our study. We made three predictions. (1) The relative calorie loss and income cost to farmers will be highest for maize compared to other damaged food crops. Maize is reported to be of higher nutritional value to crop raiding animals (Alemayehu & Tekalign 2020; Mamo et al. 2021). Shephard et al. (2019) also reported maize to be an important cash crop for most rural African subsistence farmers. (2) The potential income and relative calories lost will be highest during the dry season as compared to the wet season, because several studies reported higher levels of crop raiding during the dry season (Branco et al. 2019; Sebsibe & Yihune 2018). (3) Farmers in our study will use scarecrows more than any other mitigation measure to combat crop raiding as compared to all the other measures, as reported in many studies on crop raiding in subsistence farming (Alemayehu &Tekalign 2020; Mekonen 2020; Wiafe 2019).

Through our interactions with local stakeholders, we were aware that the local governmental authorities and conservationists in the Hluhluwe area and the communities around the reserve were concerned about crop raiding. The reserve management had assigned community liaison officers to manage the issues arising from HWC that might lead to crop loss. These issues included food insecurity, economic costs and other opportunity costs (e.g. loss of school time for children patrolling fields).

## Methods

### Study site

The study was conducted from April 2016 to March 2017 in 20 subsistence farms at Phindisweni community (S 28°00’ E 31°42’) adjacent to the Hluhluwe Game Reserve (S 28°26 E 32°09’), South Africa. The Phindisweni village is 1.81 km^2^ in extent and had a population of 2469 during the 2011 national census (StatsSA 2011). The community is characterised by homesteads with high levels of poverty (StatsSA 2016). Approximately 96% of the community members depended on crop-based agriculture for their subsistence (StatsSA 2016). The lack of reticulated water, sanitation and electricity were the most pressing issues in the community. Only one homestead reported having electricity in the 2016 community census. These farms were located mainly on hilly terrain. The natural vegetation type in this community was savannah grassland (eds. Cromsigt, Archibald & Owen-Smith 2017). Like most farming communities abutting protected areas in Africa, this community was affected by crop raiding by wildlife in the past (Infield 1986, 1988) as well as during our study.

Various crops are cultivated by subsistence farmers in the study area, including grain, leafy green vegetables, root vegetable staples and fruits. However, individual farmers generally concentrated their efforts into cultivating maize (*Zea mays*), common bean (*Phaoseolus vulgaris*), spinach (*Spinacia oleracea*) and beetroot (*Beta vulgaris*). We focussed on these four commonly grown crops that the farmers considered to be central to their subsistence (personal communication). Maize is the main source of carbohydrates and common beans are the major source of protein in the diet of the farmers and their homesteads. Farmers did not invest much time into cash crops but, instead, sold surplus of the common crops when necessary.

### Research design

A research design is a systematic plan and procedure to be followed in integrating the different components of the study in a coherent and logical manner to effectively address the research problem and answer the research questions (Leedy & Ormrod 2015). We adopted a mixed method design including both qualitative and quantitative methods for a more holistic approach (Leedy & Ormrod 2015).

To evaluate the crop damage caused by wildlife, crop samples were collected inside quadrats. We used 6–16 quadrats, depending on the size of the cultivated land in each farm. We collected the leaves of crops because these were the most prominent parts of the crops which are damaged in the case of beetroot and spinach. Whilst the beetroot tuber is consumed by some animals, no tubers were damaged during our study. Nonetheless, we collected beetroot leaves because these were commonly used for relish by the rural farmers and had nutritional and economic value to these communities. Damaged maize cobs and common bean pods were also collected.

We used a Garmin Global Positioning System (GPS) Map62 handheld device to record the geographical location (GPS coordinates points of the farms) and slope (in metres) of each of the 20 farms sampled (see below).

We sampled for macro- and micro-fauna, using a combination of direct observations, camera trap footage and live trapping for small mammals.

### Data collection

Primary data were collected on 20 subsistence farms from April 2016 to March 2017. The dataset was generated using direct field measurements to identify, collect and analyse the damaged crops. In addition, 60 semi-structured questionnaires were administered to the farmers to obtain the details of mitigation measures employed by the farmers to deter crop raiding animals, which could not be obtained from field surveys. The questionnaire survey used was adapted from another study (Raphela & Pillay 2021).

We used a stratified sampling approach to sample the homesteads. We selected every second homestead for the interview. The selection of the homesteads was performed in such a way that they were located at a maximum of 6 km from the reserve boundary. The potential interviewees were asked whether they wanted to participate in the survey and the interview proceeded only if they agreed. We restricted the survey to one respondent per homestead to avoid pseudo-replication of results. The identity of all respondents remained anonymous during this study as outlined in the conditions of our ethics permit. We gathered signed consent forms from each respondent to participate in the study before conducting each survey.

#### Direct observations

The farms were sampled for 4 h a day randomly from 06:00 to 08:00 and, again, from 16:00 to 18:00 for 10 days over a period of 12 months. This was to capture the tracks of animals that raid during the night and observe those that raid during the day. Other studies sampled farms abutting protected areas twice, in the mornings and afternoons (Hill 1997; Naughton-Treves 1998; Tweheyo, Hill & Obua 2005). We walked throughout the perimeter of the farms to identify animal feeding on crops. However, throughout the study, larger mammals never entered the farms to raid the crops. Our observations were almost always of birds and insects feeding on crops. We never observed any wild animals feeding on crops nor any large animals’ footprints in and around the farms throughout our study.

#### Camera trap surveillance

We set up 10 × 8-megapixel infrared camera traps (Bushnell®, trophy camera, China 2012) at 10 sites which are reported to be frequently visited by primates, according to farmers’ reports. The cameras were positioned at appropriate angles at approximately 0.7 m above the ground. All the cameras faced onto the farms and were secured using multiple lengths of coated flexible wire and a padlock to prevent theft. The cameras were operational 24 h per day for 10 days per month throughout the study and were checked every 3 days to replace data storage card and batteries, if necessary, and to download video footage.

#### Small mammal live-trapping

We sampled small mammals by using the capture, mark, identify and release technique (Mills et al. 1995). Trapping was performed monthly from April 2016 to March 2017. Each trapping session lasted 10 consecutive days each month per farm. Polyvinyl chloride (PVC) live-traps (290 mm × 60 mm × 80 mm) were set randomly on each farm, resulting in 1200 (smallest farm) to 1680 (largest farm) trap nights. The traps were baited with a mixture of peanut butter, oats, coarse salt, sunflower oil and raisins (Janova et al. 2011). The traps were covered with surrounding vegetation for insulation against lethal temperatures, and cotton wool was also inserted into the traps to provide insulation for trapped animals (Torre et al. 2016). These trapping procedures were acceptable, humane trapping methods (Sikes & Gannon 2011), and were approved by the animal ethics committee of the University of the Witwatersrand.

#### Crop sampling

We visited the 20 farms for 10 consecutive days, twice a day (morning and evening) every month for the duration of the study to assess the level of crop damage. Damaged crops were identified by observing teeth marks, ragged breaks with shredded edges and holes caused by rodents, round holes on the leaves by insects and tears of the food crops by birds. Crop damage was quantified by counting the total number of individual crop samples (i.e. whole leaves of beetroot, spinach and seeds of maize and common bean) in each quadrat in the 20 sampled farms and the total number of damaged crops was recorded in each sampled farm.

#### Determination of calories

Powdered crop samples were oven dried at 150 °C for 2 h in an Ecotherm^TM^ Labotech laboratory drying oven to remove moisture. Powdered crop samples were transferred to a crucible on a Sartorius balance to weigh ~ 0.5 g or less of each sample using a stainless steel laboratory spatula. The number of samples burned per individual sampling unit depended on the dry mass of each sample, and ranged from 42.16 g to 205.52 g across all the crops sampled. A fully automatic e2k combustion oxygen bomb calorimeter (Parr Instrument Company, United States [USA]) was utilised to obtain calorific values of the collected crop samples. The oxygen flow into the calorimeter was set at 300 atm. The calorific values in kJ/g were recorded to two decimal places.

#### Energy loss

We estimated the potential energy loss by multiplying the calorific values obtained from the bomb calorimetry analyses by the proportional level of damage values. Separate analyses were conducted for dry (March-August) and wet (September–February) seasons. For example, the overall potential energy loss (relative calorie loss) for beetroot during the dry season in farm #1 was 99.2 kJ/g calculated using the proportional level of damage multiplied by the calorific values obtained in our study, as follows:


Relative calorie loss=proportional level of damage×calorific value(kJ/g). [Eqn 1]

Thus, (0.2 kJ/g × 496 kJ/g) = 862.02 kJ/g

#### Income loss

To investigate the economic impact of crop raiding, we estimated the potential income loss incurred because of damage to beetroot, common bean, maize and spinach seasonally. We used the number of damaged crop types to determine the loss in monetary value. We obtained the average cost of different crop types from the street vendors and local food markets around Hluhluwe Game Reserve. The costs differed by crop type.

To determine the potential income loss, we multiplied the number of damaged crop type bunches (obtained by dividing the average number of leaves/pods per bunch by the level of damage) by the average cost value (ZAR) of these individual crops. For example, the potential income loss for beetroot during the dry season in Farm #1 was calculated as follows:


Potential income loss=Number of damaged crop type bunches×ZAR. [Eqn 2]

Thus, 2 bunches = 2 × ZAR 14.00 = ZAR 28.00

To calculate the number of maize cobs damaged in each farm, we determined the number of cobs that lost seeds and could not be sold (information provided by the farmers). The potential income loss value for maize was obtained by multiplying the number of damaged maize crops by the average cost value (ZAR) in the dry and wet seasons of maize in 2018, obtained from street vendors and local markets. The costs differed seasonally for all crop types.

The prices of beetroot, common bean, maize and spinach during the dry and wet season were obtained from street vendors and food markets a few kilometres from the Hluhluwe Game Reserve.

### Data analysis

All data were recorded in Microsoft Excel® (Microsoft Corporation 2007), and statistical analyses were conducted using R Statistical Software (www.r-project.org, R version 4.1.0, 18 May 2021). Statistical tests were two-tailed, and significance levels were set at *p* ≤ 0.05. All graphs were produced using a Grammar of Graphics (GG) plot2 package in the R software. The Spearman rank order correlation coefficient (Spearman’s rho) was performed to analyse the relationship between proximity of a farm to the reserve boundary and the level of crop raiding. The level of crop damage was set as the response variable and distance from the game reserve was the explanatory variable. Rodent trapping data were analysed using descriptive statistics because of the small sample sizes.

We used Generalised Linear Mixed Models (GLMMs, *glmer* function and a log distribution; lme4 package; Bates et al. 2015) to analyse the potential nutritional and monetary cost to farmers. We combined the data by month for all the variables (level of damage, calorific values and costs of damaged crop types) and grouped these for the dry and wet seasons.

Separate GLMM analyses were conducted for relative calorie loss and potential income loss (dependent variables). Season and the interaction between crop type and season were set as predictor variables in the analyses. For each predictor, one category was set as a reference, to which others were compared. For both models, we included farm size as a random factor (intercepts only) to account for the potential farm size effect because the relative calorie loss and potential income could be influenced by the size of the farms planted. We checked the model fit for the variables described above, and used the most appropriate model, based on the plot of the residuals against the fitted values from each model (Crawley 2012). We generated *p* values using likelihood ratio tests (Bates et al. 2015). To analyse relative calorie loss, we included the slope of the farms as a continuous predictor variable because the aspect can influence soil conditions (e.g. water, micro-nutrients) and hence calorie loss. We provided estimate coefficients, standard errors, *Z* and *p* values from the model output for potential income loss. In addition, we calculated Wald (χ^2^) statistics (*car* function) and used post-hoc pairwise differences comparisons for significant predictors (*emmeans* package), as appropriate for both models.

The choice of mitigation measures to reduce crop raiding on farms may be influenced by several factors. Therefore, we applied a multinomial logistic regression analysis fitted from the nnet package in *R* with a multinom function to investigate the relationship between mitigation measures (dependent variable) employed by the farmers and their reports of three independent variables, that is, crop raiding animal type, crop types raided and the distance of farms from the reserve boundary. These issues were considered separately during the survey.

### Ethical considerations

Ethical clearance was obtained from University of the Witwatersrand, South Africa’s Animal Ethics Screening Committee (2015/08/37/B) and Human Ethics screening committee (HREC) (non-medical) under protocol number H15/11/29.

## Results

In the 1 year of sampling, we did not record any crop raiding by large mammals, both from direct observations and camera trap footage. Large mammals (herbivores, elephants and primates) were observed in the reserve but none entered into the farmland. Instead, micro-fauna, including rodents, birds and insects were mostly responsible for the crop damage in our study. We did not find a statistically significant relationship between the level of damage and the distance of farms from the reserve boundary (Spearman’s rho rs = –0.07, *p* = 0.438).

A total of 96 individual rodents were captured in 20 sampled farms from April 2016 to March 2017 in 30 600 trap nights (0.3% trap success), comprising two species, namely the red bush rat (*Aethomys* spp.) and the pouched mouse (*Saccostomus campestris*). *Aethomys* spp. (67.7%; 51 males and 28 females) was most commonly trapped, and is a common murid rodent in the savanna habitats of KwaZulu-Natal Province (McGuinness & Taylor 2014). The pouched mouse (*Saccostomus campestris*) represented the remaining 32.3% (14 females and three males). Both the *Aethomys* spp. and the *Saccostomus campestris* were mostly captured during the dry season.

### Variations in relative calorie loss

**FIGURE 1:**
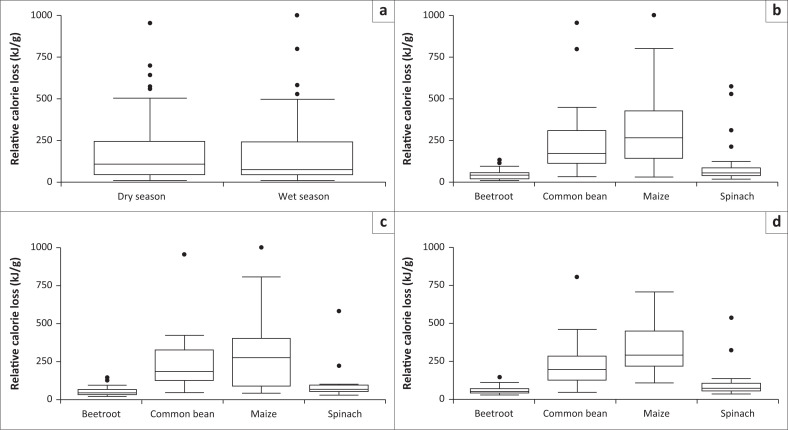
Relative calorie loss by season (a), crop type (b) and the interaction between season and crop type (c [dry season] and d [wet season]) experienced by farmers on the edge of Hluhluwe Game Reserve, South Africa. Boxes show 1st and 3rd quartiles and medians (solid black line across the box). Whiskers show total range and dots outside of boxes indicate outliers.

Season (Wald χ^2^_1_ = 11.256, *p* = 0.000), crop type (Wald χ^2^_3_ = 8836.07, *p* < 0.001), farm slope (Wald χ^2^_1_ = 26.40, *p* < 0.001) and the interaction between season and crop type (Wald χ^2^_3_ = 26.77, *p* < 0.001) were significant predictors of relative calorie loss. Significantly higher relative calorie loss occurred during the dry season as compared to the wet season. The highest calorie loss was for maize followed by common bean, spinach and beetroot, respectively. In addition, the highest relative calorie loss recorded in the dry season was for maize (Figure 1).

### Seasonal variations in potential income losses by crop type

**FIGURE 2:**
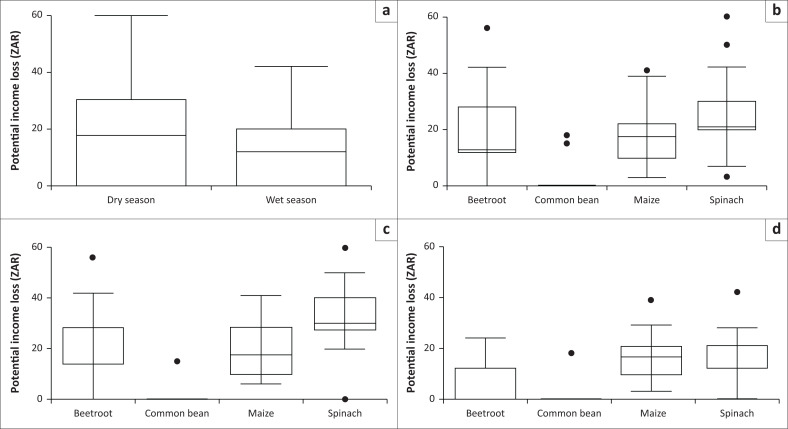
Potential income loss by season (a), crop type (b) and the interaction between season and crop type (c [dry season] and d [wet season]) experienced by farmers on the edge of Hluhluwe Game Reserve, South Africa. Boxes show 1st and 3rd quartiles and medians (solid black line across the box). Whiskers show total range and dots outside of boxes indicate outliers.

Season (Wald χ^2^_1_ = 165.40, *p* < 0.001), crop type (Wald χ^2^_3_ = 388.38, *p* < 0.001) and the interaction between season and crop type (Wald χ^2^_3_ = 63.33, *p* < 0.001) were significant predictors of the potential income loss. Significantly higher potential income loss was incurred during the dry season as compared to the wet season (Figure 2). The lowest income loss was for common bean, followed by beetroot, maize and spinach (Figure 2). The highest potential income loss was greater for spinach, beetroot and maize in the dry season as compared to the wet season, but maize and common bean income loss did not differ seasonally (Figure 2).

Furthermore, there were significant differences found for potential income loss for all crop types, the wet season and for the interaction between the crop types maize, spinach and the wet season. But no significant difference was found for crop type common bean and the wet season (Table 1).

**TABLE 1:** Output of a generalised linear mixed models for comparisons of crops damaged for potential income loss.

Variables	Estimate	Standard error	*Z*	*p*
Crop type_common bean	3.099e+00	2.621e–01	–13.299	< 0.001*
Crop type_maize	–3.486e+00	6.801e–02	–3.470	0.000*
Crop type_spinach	–2.360e–01	5.945e–02	5.263	< 0.001*
Wet season	3.129e–01	8.716e–02	–11.489	< 0.001*
Crop type_common bean: Wet season	–2.204e+01	2.601e+04	–0.001	0.999
Crop type_maize: Wet season	8.145e–01	1.153e–01	7.063	< 0.001*
Crop type_spinach: Wet season	3.913e–01	1.088e–01	3.597	0.000*

* , indicate significant values.

All sampled farms experienced some level of damage for all or most crops, and based on the calculation of the potential income loss used in this study, the potential income loss ranged from ZAR 0.00 to ZAR 60.00. Overall, the potential income ranged from ZAR 0.00 to ZAR 60.00 for spinach, ZAR 0.00 to ZAR 56.00 for beetroot, ZAR 0.00 to ZAR 41.00 for maize and ZAR 0.00 to ZAR 18.00 for common bean in both seasons.

Using the data in Online Appendix, Table 1-A1, we calculated that the potential income loss for all farms per annum as ZAR 2427.00 (*about US$150 at an exchange rate of ZAR 16.18*) at an average of ZAR 77.35 (standard deviation [s.d.] = ZAR 123.83) per farm in the dry season as compared to a mean of ZAR 44.00 (s.d. = ZAR 71.179) in the wet season.

### Mitigation measures

**FIGURE 3:**
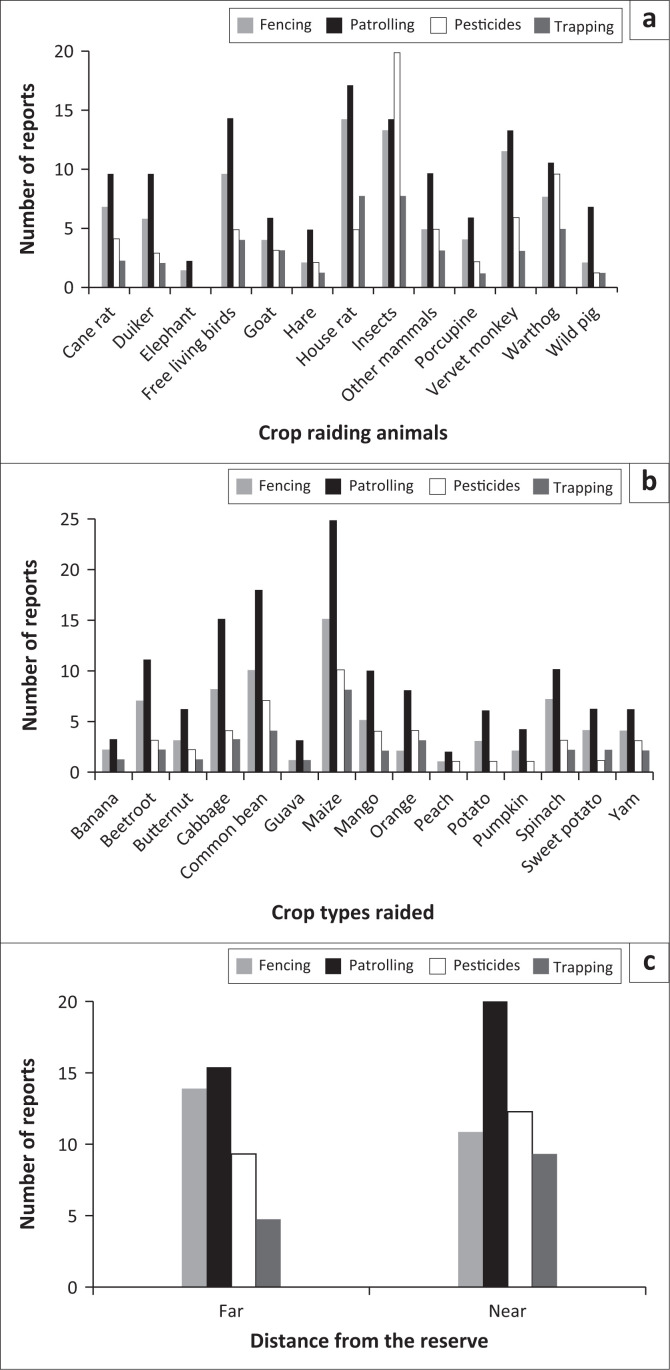
The number of reports about the mitigation measures (fencing, patrolling, pesticides and trapping) used by farmers for crop raiding animals (a), for crop types damaged (b), by the distance (c) of farms from the edge of Hluhluwe Game Reserve, South Africa.

All respondents reported using several different types of mitigation measures (i.e. patrolling, fencing, pesticides and trapping) to deter crop-raiding animals on their farms. We used a multinomial logistic regression to assess the combined relationship of control measures versus crop raiding animals, crop types raided and distance from the reserve boundary. The analysis revealed that crop raiding animal types (Wald χ^2^_4_ = 18.49, *p* = 0.000), crop types raided (Wald χ^2^_4_ = 66.91, *p* < 0.001) and distance of farms from the reserve boundary (Wald χ^2^_4_ =10.69, *p* = 0.030) all had a significant effect on the choice of mitigation measures used by the farmers. Overall, more farmers chose patrolling compared to fencing, pesticides and trapping, but pesticides were chosen more than patrolling, fencing and trapping for crop raiding animals, insects in particular (Figure 3). However, to protect maize, farmers used patrolling more than any other control measure. The majority of farmers near and far away from the reserve still chose patrolling to deter the crop raiding animals (Figure 3).

## Discussion

We investigated the seasonal calorie loss and potential economic impact because of crop raiding on subsistence farming homesteads abutting the Hluhluwe Game Reserve, and the mitigation measures employed by farmers to combat crop raiding. Our intention was to investigate the crop raiding by macro- and micro-fauna. However, we did not find any indications of crop damage by macro-fauna during our study. Thus, we recorded the crop damage by micro-fauna only. However, there were historical reports of macro-fauna raiding crops in the study area (Infield 1988).

Consistent with our two predictions, we found the highest relative calorie loss and income loss in the dry season, recorded for maize as compared to other food crops. Indeed, several other studies reported greater levels of crop damage and nutrient loss in the drier seasons (Chiyo et al. 2005; Dudley, Mensah-Ntiamoah & Kpelle 1992; Naughton-Treves 1998; Nyhus & Tilson 2000; Rode et al. 2006). Similarly, during the dry season, the frequency of crop raiding was related to the loss of higher nutrient contents of cultivated crops at Way Kambas National Park in Sumatra (Nyhus & Tilson 2000).

There may be two explanations for the greater relative calorie loss for maize compared to other crop types. Firstly, the level of damage for maize was highest compared to other crops in our study. Therefore, the higher relative calorie loss for maize is most likely attributable to the higher levels of damage. Secondly, previous research has reported that maize is high in nutrients (Alemayehu & Tekalign 2020; Koirala et al. 2021), and has higher energy than other crops (Schley & Roper 2003). Mekonen (2020) reported that maize was the most frequently eaten crop by humans and animal crop raiders in West Africa because of its nutritional value.

The highest potential income loss was during the dry season. This was not surprising because the highest level of crop damage occurred during the dry season, and the prices of crops were higher in the dry season rather than the wet season. The literature on seasonal variation in the economic impact of crop raiding on subsistence farming communities is limited, and instead annual loss is reported. We calculated a potential income loss of ZAR 2427.00 (*about US$150*) per annum for all crop types combined. This amount appears trivial from an international perspective but it is a significant amount (about 17% of total income) in an area where homestead income average is ZAR 15 000.00 per annum (US$ 1116.90; StatsSA 2011). We found the highest potential income loss to be for spinach, despite the highest level of damage being for maize. This was surprising considering the low street vendor cost and the lowest level of damage of spinach crop as compared to other crops. This finding is also in contrast to many other studies that reported higher potential income cost to farmers for maize because of crop damage (Heinen 1993; Naughton-Treves 1998; Newmark et al. 1994; Parry & Campbell 1992; Studsrod & Wegge 1995). However, most of these studies have quantified the damage based on questionnaire surveys rather than measurement of the level of crop damage directly, as in our study.

Crop raiding animal types, crop types raided and the distance of farms from the reserve boundary all had significant effects on the choice of mitigation measures which the farmers used. However, patrolling of fields was the most prominent mitigation measure employed by farmers in our study to deter crop raiders and protect raided crops. Our findings suggest that the farmers around the Hluhluwe Game Reserve should invest in the mitigation measures targeted mostly at micro-fauna, especially insects, which were reported to be most damaging fauna. The use of pesticides reported by the farmers to deter crop raiding insects might have an adverse effect on the farmers’ health in the future. Maize was the most protected crop. This was not surprising because maize is reported to be a food security crop especially in South Africa (Sinyolo 2020), leading to physical guarding of this crop. But, this might have future problems because mostly children patrolled the fields, so there might be opportunity costs, such as loss of school time for the children. This will exacerbate the poverty faced by this community because it may lead to future difficulties related to finding a decent job by the people.

## Conclusion

In conclusion, our findings suggest that crop loss by micro-fauna could reduce annual calorie intake and incur potential income loss of farming homesteads adjacent to the Hluhluwe Game Reserve. Our results can be used by the local government authorities to assess the plight of the subsistence farming community in our study area. In addition, the Hluhluwe Game Reserve management can be made aware of the plight of the farming community because of crop loss by micro-fauna, without the impact of macro-fauna, whose influence could be far more substantial and hence needs to be carefully scrutinised by the reserve management.

### Recommendations

It is difficult to predict whether a lack of crop raiding by larger mammals was because of environmental (drought) conditions or effective deterrents by farmers and/or effective barriers being erected by the reserve management. We therefore recommend that future studies of crop raiding must be conducted over longer periods under various environmental conditions to assess the conditions under which macro-fauna crop raid in our study site and whether or not the community is affected by this crop-raiding. We also recommend that the farmers, with the assistance of the reserve management, invest in relevant mitigation measures to reduce the impact of crop raiding by micro-fauna.
